# Supplementary material for: Comparison and Identification for Rhizomes and Leaves of Paris yunnanensis Based on Fourier Transform Mid-Infrared Spectroscopy Combined with Chemometrics
Source: Molecules. 2018 Dec 17;23(12):3343. doi: 10.3390/molecules23123343 (PMC6320853; doi:10.3390/molecules23123343)
Supplement: Supplementary file 1 [file molecules-23-03343-s001.pdf]

Comparison and identification for rhizomes and leaves of *Paris yunnanensis* based on Fourier transform mid infrared spectroscopy combined with chemometrics

Yi-Fei Pei [a, b], Qing-Zhi Zhang [b], Zhi-Tian Zuo [a*] and Yuan-Zhong Wang [a*]

[a] Institute of Medicinal Plants, Yunnan Academy of Agricultural Sciences, Kunming 650200, PR China.

[b] College of Traditional Chinese Medicine, Yunnan University of Traditional Chinese Medicine, Kunming 650500, PR China.

*Corresponding author:

Mr Yuan-Zhong Wang and **Mrs Zhi-Tian Zuo**, Institute of Medicine Plants, Yunnan Academy of Agricultural Science, 2238, Beijing Road, Panlong District, Kunming 650200, China. Tel: +86 871-65033575, Fax: +86 871-65033441, E-mail: boletus@126.com (**Mr Yuan-Zhong Wang**), yaaszztian@126.com (**Mrs Zhi-Tian Zuo**)

Figure captions

Fig. S1 VIP scores of FT-MIR data of leaves for regional difference: (a) raw dataset, (b) SNV-SD dataset.

Fig. S2 The n_{tree} and m_{try} screening of RF models of *P. yunnanensis* samples before variables ranked by permutation accuracy importance: (a) n_{tree} of raw leaves dataset, (b) n_{tree} of SNV-SD leaves dataset, (c) m_{try} of raw leaves dataset, (d) m_{try} of SNV-SD leaves dataset.

Fig. S3 The 10-fold cross validation error rates of RF model (sequentially reduce each five variables) based on *P. yunnanensis* samples: (a) raw leaves dataset, (b) SNV-SD leaves dataset.

Fig. S4 The n_{tree} and m_{try} screening of RF models of *P. yunnanensis* samples after variables ranked by permutation accuracy importance: (a) n_{tree} of raw leaves dataset, (b) n_{tree} of SNV-SD leaves dataset, (c) m_{try} of raw leaves dataset, (d) m_{try} of SNV-SD leaves dataset.

Fig. S5 The n_{tree} and m_{try} screening of RF models of *P. yunnanensis* samples before variables ranked by permutation accuracy importance: (a) n_{tree} of raw data fusion dataset, (b) n_{tree} of SNV-SD data fusion dataset, (c) m_{try} of raw data fusion dataset, (d) m_{try} of SNV-SD data fusion dataset.

Fig. S6 The 10-fold cross validation error rates of RF model (sequentially reduce each five variables) based on *P. yunnanensis* samples: (a) raw data fusion dataset, (b) SNV-SD data fusion dataset.

Fig. S7 The n_{tree} and m_{try} screening of RF models of *P. yunnanensis* samples after variables ranked by permutation accuracy importance: (a) n_{tree} of raw data fusion dataset, (b) n_{tree} of SNV-SD data fusion dataset, (c) m_{try} of raw data fusion dataset, (d) m_{try} of SNV-SD data fusion dataset.

Table captions

Table S1 The major parameters of raw and preprocessing calibration models based on *P. yunnanensis* samples combined with rhizomes FT-MIR spectra.

Table S2 The major parameters of PLS-DA and RF models of each class based on raw and SNV-SD rhizomes FT-MIR spectra datasets of *P. yunnanensis* samples.

Table S3 The major parameters of raw and preprocessing calibration models based on *P. yunnanensis* samples combined with leaves FT-MIR spectra.

Table S4 The major parameters of PLS-DA and RF models of each class based on raw and SNV-SD leave FT-MIR spectra datasets of *P. yunnanensis* samples.

Table S5 The geographical location of *P. yunnanensis* samples.

Table S6 The sample size of calibration set and validation set for each class.

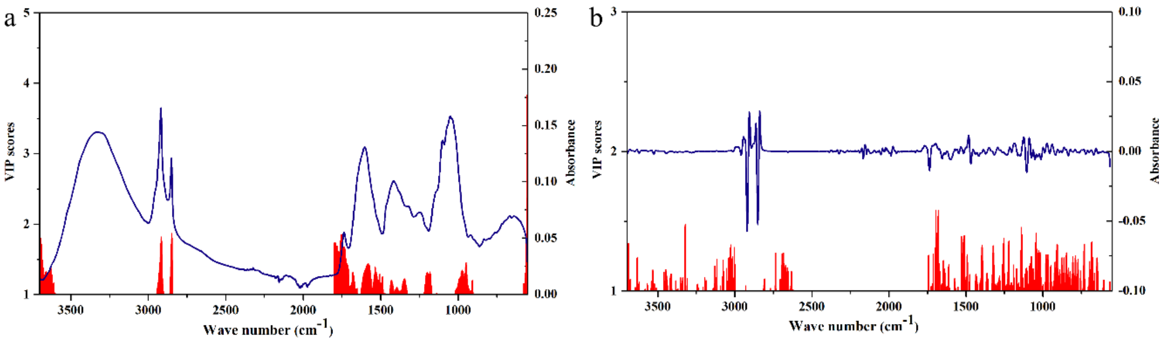
a
b
VIP scores
Absorbance
Wave number (cm^{-1})
3500
3000
2500
2000
1500
1000
0.25
0.20
0.15
0.10
0.05
0.00
-0.05
-0.10

Fig. S1

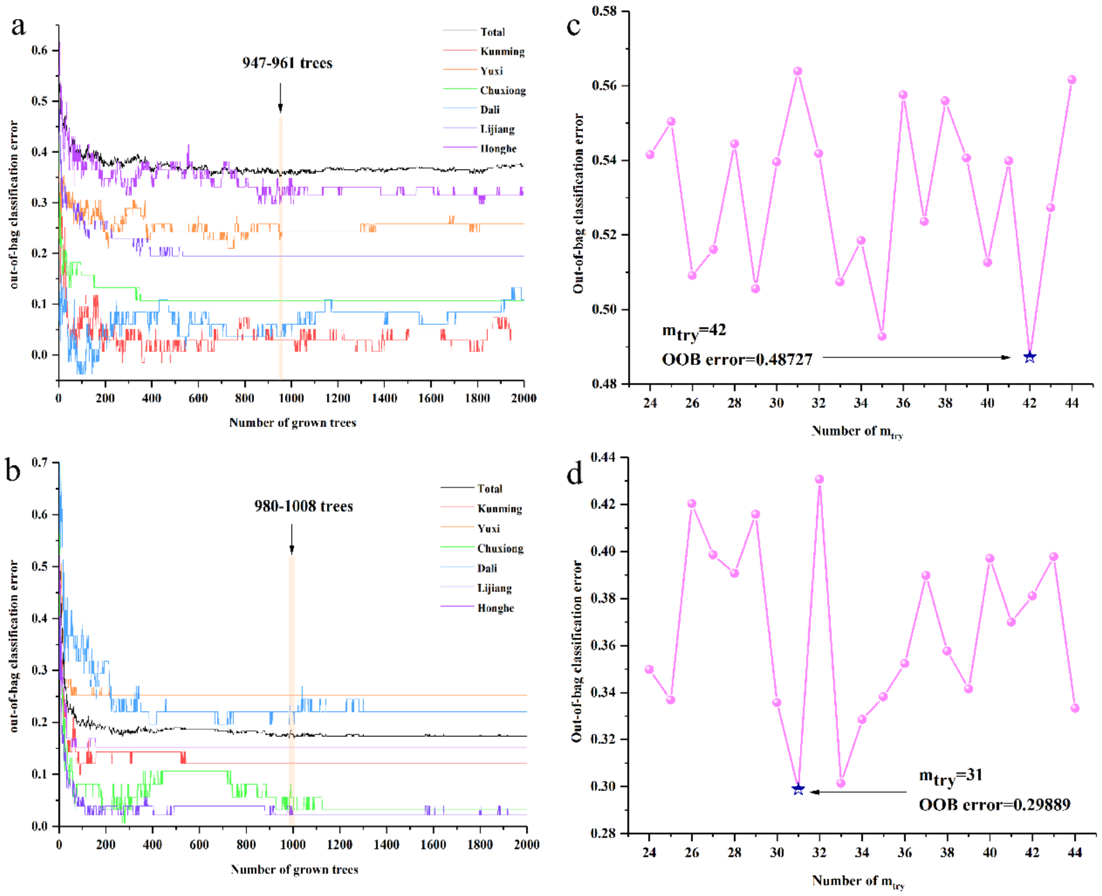

a
947-961 trees
Total
Kunming
Yuxi
Chuxiong
Dali
Lijiang
Honghe
out-of-bag classification error
Number of grown trees
c
Out-of-bag classification error
m_{try}=42
OOB error=0.48727
Number of m_{try}
b
980-1008 trees
Total
Kunming
Yuxi
Chuxiong
Dali
Lijiang
Honghe
out-of-bag classification error
Number of grown trees
d
Out-of-bag classification error
m_{try}=31
OOB error=0.29889
Number of m_{try}

Fig. S2

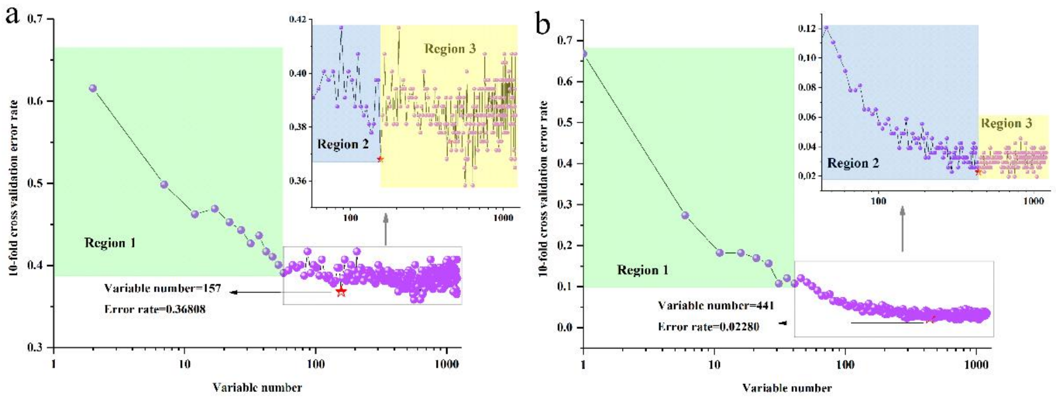

a
b
Region 1
Region 2
Region 3
Variable number=157
Error rate=0.36808
Variable number=441
Error rate=0.02280
10-fold cross validation error rate
Variable number

Fig. S3

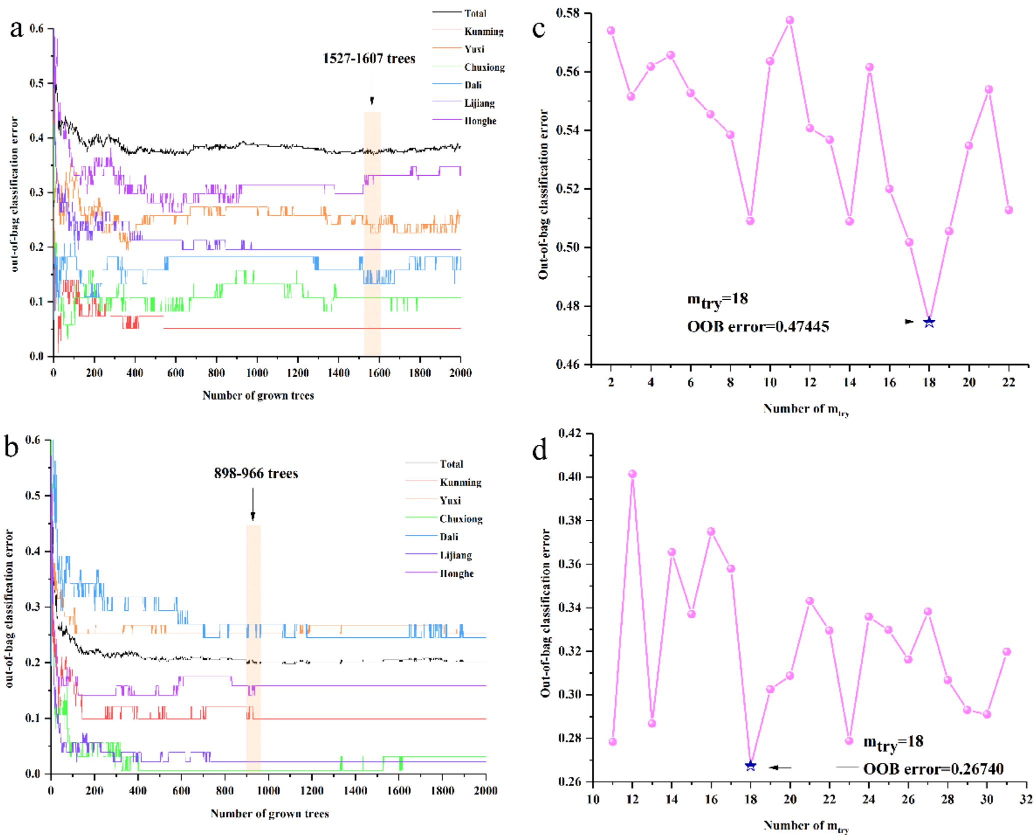

a
1527-1607 trees
Total
Kunming
Yuxi
Chuxiong
Dali
Lijiang
Honghe
out-of-bag classification error
Number of grown trees
b
898-966 trees
c
m_{try}=18
OOB error=0.47445
Out-of-bag classification error
Number of m_{try}
d
m_{try}=18
OOB error=0.26740

Fig. S4

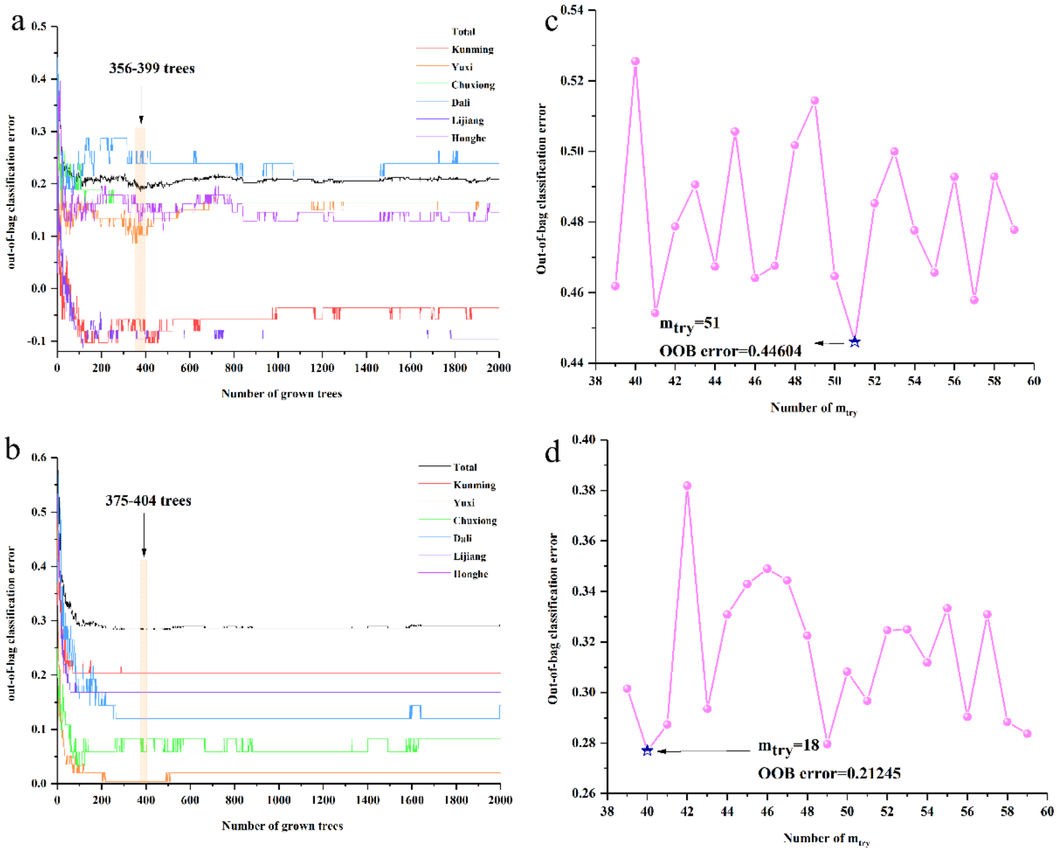
a
356-399 trees
out-of-bag classification error
Number of grown trees
Total
Kunming
Yuxi
Chuxiong
Dali
Lijiang
Honghe
b
375-404 trees
out-of-bag classification error
Number of grown trees
Total
Kunming
Yuxi
Chuxiong
Dali
Lijiang
Honghe
c
Out-of-bag classification error
m_{try}=51
OOB error=0.44604
Number of m_{try}
d
Out-of-bag classification error
m_{try}=18
OOB error=0.21245
Number of m_{try}

Fig. S5

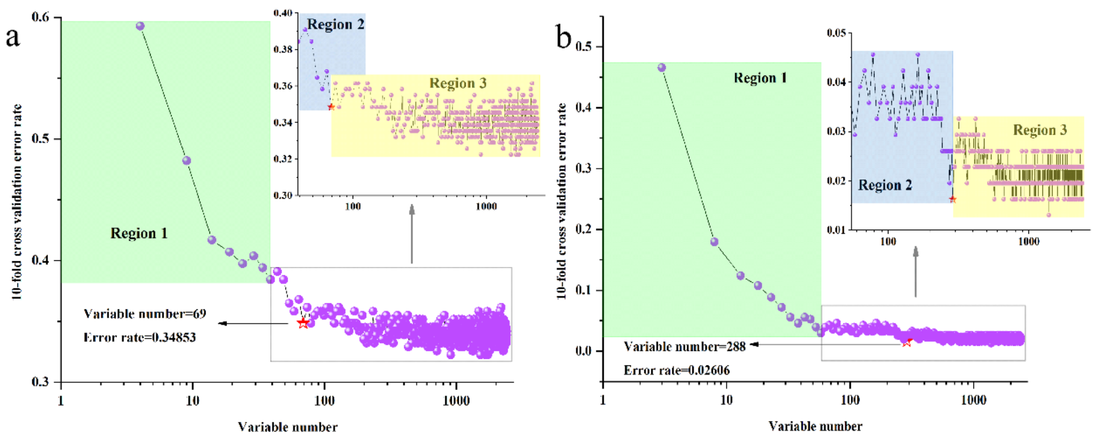
a
10-fold cross validation error rate
0.6
0.5
0.4
0.3
Region 1
Region 2
Region 3
Variable number=69
Error rate=0.34853
1
10
100
1000
Variable number
b
Region 1
Region 2
Region 3
Variable number=288
Error rate=0.02606
10-fold cross validation error rate
Variable number

Fig. S6

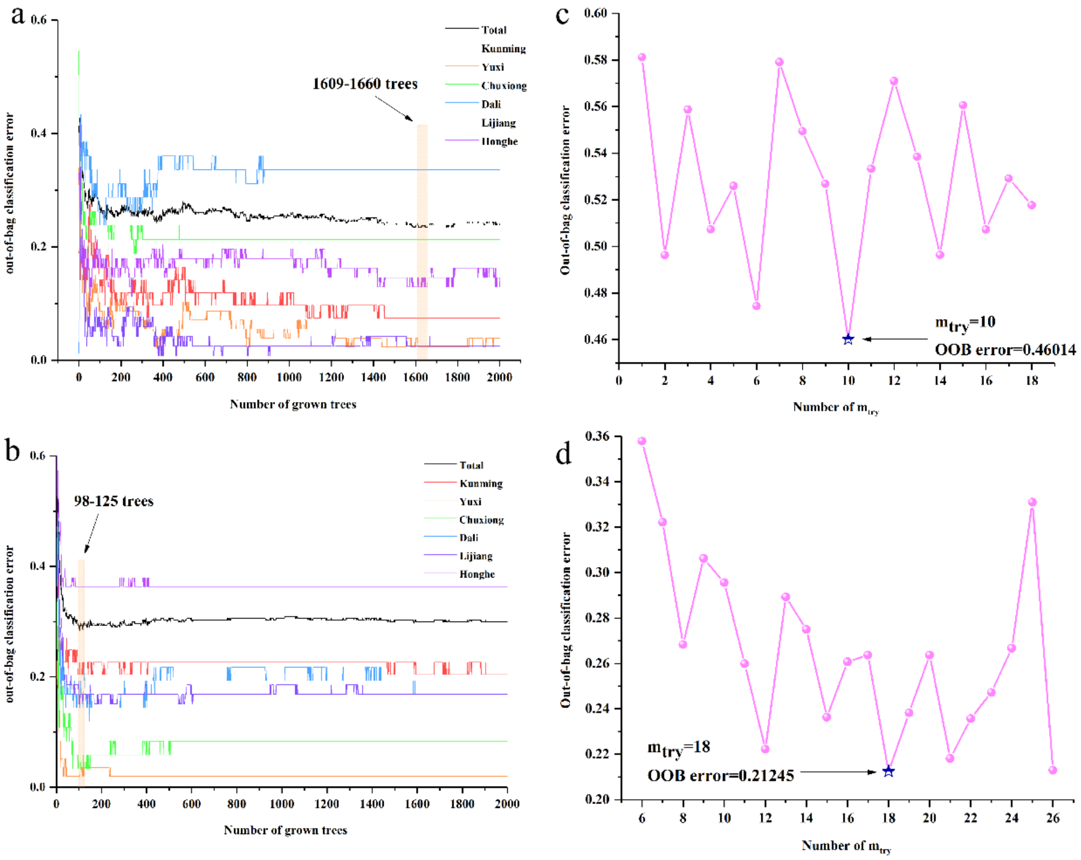
a
b
c
d
out-of-bag classification error
Number of grown trees
Total
Kunming
Yuxi
Chuxiong
Dali
Lijiang
Honghe
1609-1660 trees
98-125 trees
Out-of-bag classification error
Number of m_{try}
m_{try}=10
OOB error=0.46014
m_{try}=18
OOB error=0.21245

Fig. S7

Table S1

Preprocessing	LVs	R^2	Q^2	RMSEE	RMSECV	Accuracy
Raw	18	0.714	0.584	0.201239	0.250368	95.44%
SNV	18	0.696	0.56	0.208316	0.254995	95.77%
SNV-FD	19	0.775	0.614	0.179515	0.239488	99.02%
SNV-SD	14	0.816	0.674	0.162205	0.219739	99.67%
SD	16	0.824	0.63	0.174812	0.237413	100%

Table S2

Preprocessing	Set	Classes [a]	PLS-DA				RF			
			SENS	SPEC	ACC	MCC	SENS	SPEC	ACC	MCC
Raw	Calibration set	1	1	1	1	1	0.6	0.962	0.909	0.61
		2	0.968	0.992	0.987	0.96	0.698	0.955	0.902	0.688
		3	0.775	1	0.971	0.866	0.625	0.974	0.928	0.66
		4	0.927	0.992	0.984	0.929	0.561	0.951	0.899	0.541
		5	1	0.972	0.977	0.931	0.69	0.9	0.86	0.565
		6	1	0.988	0.99	0.97	0.764	0.861	0.844	0.553
	Validation set	1	1	0.992	0.994	0.975	0.87	0.977	0.961	0.847
		2	0.75	0.992	0.942	0.816	0.469	1	0.89	0.642
		3	0.619	1	0.948	0.764	0.238	1	0.897	0.461
		4	0.95	1	0.994	0.971	0.8	1	0.974	0.881
		5	1	0.937	0.948	0.857	0.931	0.849	0.865	0.666
		6	1	0.944	0.955	0.875	0.9333	0.824	0.845	0.64
SNV-SD	Calibration set	1	1	1	1	1	0.933	0.992	0.984	0.934
		2	1	1	1	1	0.921	0.996	0.98	0.939
		3	0.975	1	0.997	0.986	0.8	0.989	0.964	0.835
		4	1	1	1	1	0.854	0.981	0.964	0.844
		5	1	0.996	0.997	0.989	0.897	0.948	0.938	0.809
		6	1	1	1	1	0.967	0.976	0.974	0.92
	Validation set	1	1	1	1	1	1	1	1	1
		2	1	1	1	1	0.969	1	0.994	0.98
		3	0.905	1	0.987	0.944	0.905	1	0.987	0.944
		4	1	0.993	0.994	0.972	0.85	0.993	0.974	0.882
		5	1	1	1	1	0.966	0.96	0.961	0.882
		6	1	0.992	0.994	0.98	1	0.992	0.994	0.98

Table S3

Preprocessing	LVs	R^2	Q^2	RMSEE	RMSECV	Accuracy
Raw	18	0.698	0.559	0.203855	0.203855	95.77%
SNV	17	0.719	0.549	0.198488	0.256876	97.72%
SNV-FD	17	0.807	0.622	0.165141	0.240036	100%
SNV-SD	15	0.876	0.754	0.133634	0.195234	100%
SD	14	0.837	0.704	0.151242	0.210209	99.67%

Table S4

Preprocessing	Set	Classes [a]	PLS-DA				RF			
			SENS	SPEC	ACC	MCC	SENS	SPEC	ACC	MCC
Raw	Calibration set	1	0.978	0.989	0.987	0.949	0.556	0.924	0.87	0.479
		2	0.984	1	0.997	0.99	0.667	0.922	0.87	0.596
		3	1	0.978	0.98	0.922	0.85	0.963	0.948	0.781
		4	0.805	0.996	0.971	0.868	0.244	0.91	0.821	0.167
		5	0.966	0.992	0.987	0.957	0.793	0.912	0.889	0.664
		6	0.983	0.996	0.993	0.979	0.55	0.911	0.84	0.477
	Validation set	1	0.957	1	0.994	0.974	0.696	0.985	0.942	0.755
		2	1	0.984	0.987	0.962	0.938	0.967	0.961	0.885
		3	1	0.97	0.974	0.903	1	0.985	0.987	0.948
		4	0.75	0.993	0.961	0.818	0.75	0.933	0.91	0.633
		5	0.931	0.992	0.981	0.936	0.862	0.952	0.935	0.794
		6	1	1	1	1	0.767	0.984	0.942	0.806
SNV-SD	Calibration set	1	1	1	1	1	0.978	0.989	0.987	0.949
		2	1	1	1	1	0.984	0.988	0.987	0.961
		3	1	1	1	1	0.975	1	0.997	0.986
		4	1	1	1	1	0.878	0.992	0.977	0.899
		5	1	1	1	1	1	1	1	1
		6	1	1	1	1	0.983	0.996	0.993	0.979
	Validation set	1	1	1	1	1	1	1	1	1
		2	1	1	1	1	1	1	1	1
		3	0.952	1	0.994	0.972	1	1	1	1
		4	1	0.993	0.994	0.972	0.95	1	0.994	0.971
		5	1	1	1	1	1	0.992	0.994	0.979
		6	1	1	1	1	1	1	1	1

Table S5

Region	Location	Sample size	Latitude (°N)	Longitude (°E)
1	Wuhua, Kunming	68	25.042165	102.704412
2	Hongta, Yuxi	95	24.43105	102.44098
3	Yaoan, Chuxiong	61	25.522293	101.375931
4	Weishan, Dali	61	25.307049	100.316085
5	Gucheng, Lijiang	87	26.874046	100.190409
6	Yuanyang, Honghe	90	23.007286	103.025416

Table S6

	Class 1	Class 2	Class 3	Class 4	Class 5	Class 6
Calibration set	45	63	40	41	58	60
Validation set	23	32	21	20	29	30
Total	68	95	61	61	87	90
